# Foliar sprays of citric acid and salicylic acid alter the pattern of root acquisition of some minerals in sweet basil (*Ocimum basilicum* L.)

**DOI:** 10.3389/fpls.2014.00573

**Published:** 2014-10-31

**Authors:** Noushin Ghazijahani, Ebrahim Hadavi, Byoung R. Jeong

**Affiliations:** ^1^Microbiology Department, Karaj Branch, Islamic Azad UniversityKaraj, Iran; ^2^Horticulture Department, Karaj Branch, Islamic Azad UniversityKaraj, Iran; ^3^Horticulture Department, Gyeongsang National UniversityJinju, South Korea

**Keywords:** citric acid, hydroponic production, organic acids, salicylic acid, silicon, boron, sulfur

## Abstract

The effect of foliar application of two levels of citric acid (CA; 0 and 7 mM) and two levels of salicylic acid (SA; 0 and 1 mM) combined with two levels of nutrient solution strength (full strength and half strength) on mineral acquisition by sweet basil were investigated. The experiment was conducted in a randomized block design arrangement with three replications. SA alone reduced the plant height and thickened the stem. Plants supplied with a full strength solution had a ticker stem, produced more biomass, and showed higher values of Fv/Fm. Some changes in the uptake pattern of some nutrients, especially boron and sulfur, were noticed. Higher boron concentrations in leaves were in plants sprayed with a combination of 7 mM CA and 1 mM of SA. Applying combination of CA and SA was more effective than using them individually that suggests an effective synergism between them.

## INTRODUCTION

Sweet basil (*Ocimum basilicum* L.) is an herb belonging to the Lamiaceae family that is used as a favorite fresh vegetable together with many traditional foods, dressings, and salads. The aroma of basil makes an essential contribution to its organoleptic quality as well as the medicinal value.

Salicylic acid (SA) is one of the most readily available plant growth regulating materials, which is also effective in other forms of acetyl SA and methyl salicylate in the plant (Raskin, 1992). SA acts as a stress messenger that induces the hypersensitive response (HR) in the plant, helping the plant to resist invading organisms (Klessig and Malamy, 1994), and interactions between SA and other natural stress management compounds have been reported (Cipollini et al., 2004; Metraux and Durner, 2004; Kachroo and Kachroo, 2007; Zottini et al., 2007; De Torres Zabala et al., 2009). SA could induce the alternative oxidase enzyme activity in mitochondria that are involved in the stress alleviation mechanism (Raskin, 1992; Vanlerberghe and McIntosh, 1997). In lower concentrations (less than 1 mM) it has been reported to be beneficial for plant growth (Rivas-San Vicente and Plasencia, 2011). Enhancement (Kiddle et al., 1994; Kang et al., 2004; Wang et al., 2007) or decline (Prithiviraj et al., 2005; D’Onofrio et al., 2009) in specific secondary metabolites of the plant by SA was also reported. There is a report on increasing the content of essential oils of the sweet basil in response to sprays of SA at 0.1 mM (Gharib, 2006).

Citric acid (CA) is a six carbon organic acid, having a central role in CA cycle in mitochondria that creates cellular energy by phosphorylative oxidation reactions. It is created by addition of acetyl-CoA to oxaloacetic acid that is converted to succinate and malate in next steps (Wills et al., 1981). It has been documented that the exudation of citrate and malate from roots of calcicole plants (plants growing in alkaline soils) enables them to extract P and Fe from such soils (Lopez-Bucio and Nieto-Jacobo, 2000). Foliar sprays of CA alone or in combination with Fe sources have been used to recover many plants from the iron chlorosis (Mengel et al., 1994; Tagliavini et al., 1995, 2000; Abadía et al., 2002; Álvarez-Fernández et al., 2004; Eidyan et al., 2014). Later studies revealed that the CA effect is not just due to pH change and there are a variety of physiological responses to applied CA. Use of CA alone or in combinations with SA and malic acid increased the essential oil production of sweet basil (Jaafari and Hadavi, 2012) and dill (Jafari and Hadavi, 2012). Results on the application of CA, on some physiological parameters in Tuberose (Eidyan et al., 2014), *Lilium* (Darandeh and Hadavi, 2012), and Bean (El-Tohamy et al., 2013) were promising. Foliar pre-harvest application of the combinations of SA and CA in the soilless culture increased the vase life of cut rose flowers (Hajreza et al., 2013). A recent study on sweet basil revealed that the combination of 1 mM SA with 7 mM CA was superior to others in many physiological traits and yield (Mirzajani, 2013).

The ratio of variable to maximum fluorescence (Fv/Fm) represents the maximum quantum yield of the photosynthetic system II (PSII; Schreiber et al., 1995). It is a measure of efficiency of the energy conversion of PS II (Liu et al., 2011) and therefore is often considered as an index for photosynthetic efficiency (Ralph et al., 1998; Thomson et al., 2003). Fv/Fm is often used as a useful parameter to estimate the extent of photoinhibition of photosynthesis as well (Misra et al., 2012). It has been also used to check for the effect of foliar SA on efficiency of photosynthetic apparatus (Bastam et al., 2013). We used this measurement to investigate possible effects on photosynthetic efficiency as a possible mechanism of reported effects by these compounds.

The presence of a casual relationship between foliar sprays containing CA and nutrient acquisition by roots was not investigated before. Studies in soil could be biased by the effect of microorganism, which could affect on nutrient acquisition and on the other hand are dependent to organic acids excreted by roots. Here, by use of soilless system we tried to highlight the pure effect of foliar sprays on the root behavior. The present experiment was designed to assess the effect of those promising combinations of SA and CA suggested by Mirzajani (2013) on function and efficiency of nutrient absorption by the root in a hydroponic culture system. As the requirements of this plant regarding the hydroponic media strength was not studied before so we used the medium with full and half strength to create a better picture regarding the response of basil plant in a soilless culture system for use in future studies. Additionally, this enables us to study the consistency of the effect of applied combinations in different nutrient strengths.

## MATERIALS AND METHODS

This study was conducted in the experimental greenhouse of Horticulture Department at Gyeongsang National University, Jinju, South Korea during the fall season of 2013. The greenhouse climate was controlled using a fully automated system without any artificial lighting system. The mean daily temperature was 22.4°C and the mean relative humidity level was 74.7%.

The seeds of sweet basil “Karaj” were planted in plug trays on Oct 1, 2013 and the plantlets were transplanted into 10 cm plastic containers containing commercial medium (Tosilee Medium, Shinan Grow Co., Jinju, Korea) on Nov 7, while they had two full size leaves and the subsequent pair of leaves were developing. They were placed on trays of an ebb and flow system, which were irrigated twice a day. The nutrient solution (200 L tank per replication) was prepared based on the Gib’s formula (Gibeaut et al., 1997), and was amended with 1 mM (NH_4_)_2_SO_4_ as recommended by Cramer (2008).

Starting on Nov 15, the plants were sprayed manually using a 500 mL hand sprayer on a weekly basis around 12:00 am for a total of five times with combinations of CA (0 or 7 mM) and SA (0 or 1 mM), combined along with two levels of nutrient solution strength (full vs. half strength of macro elements). The 200 mM stock solutions of organic acids were used to make up the desired concentrations before spraying. As the surfactant, tween 20 (0.1 ml/L) was added to all spray solutions.

The experiment was conducted in a randomized block design factorial arrangement (2 × 2 × 2) with three replications. On Dec 16, the plants were harvested and the collected plant materials (nine plants from each replication) were weighted to estimate the fresh biomass, the plant height, and the stem diameter. The stem diameter was measured at the plant crown. The plants were then put in paper envelopes and dried in a ventilated dry oven at 70°C for 72 h after which they were weighted and the dry biomass was recorded.

The mineral contents of the leaf were measured at 3 days before harvest. We created a collective sample of 1 g fresh leaf samples by cutting out the area excluding the major veins from all nine plants in a replication. The samples were digested in 3 mL H_2_O_2_ and then added 5 mL HClO, and put in a rotary incubator overnight. The next day the samples were added with deionized water up to 50 mL and then filtered through Watman paper, and the resulting extract was analyzed for levels of inorganic nutrients. The nutrient solutions were directly sampled for analyses. An ICP-OES spectrometer (Optima 4300DV/5300DV, Perkin Elmer Inc., Waltham, MA, USA) was used to measure the levels of inorganic nutrients following the methods described by Oh et al. (2014).

The chlorophyll fluorometry was carried out with a pulse-amplitude-modulation (PAM) fluorometer (PAM-2100, H. Walz, GmbH, Effeltrich, Germany). Three measurements in each replication were conducted and averaged.

The data collected were analyzed for statistical significances with the SPSS (version 16.0, SPSS Inc., Chicago, IL, USA).

## RESULTS AND DISCUSSION

The “Results and Discussion” sections have been combined in this manuscript and so will be dealt with together in this section.

The plant height was significantly suppressed in response to 1 mM SA applied alone in both nutrient strength levels, while others were not significantly different as compared to that of the control (**Table 1**). This is in accordance with report by Nazar et al. (2011) on inhibitory effects of SA in this concentration. Both the sulfur (S) concentration and accumulation ratio in plant tissue were significantly higher in the treatment with spray of 1 mM SA on plants grown in a half-strength medium (**Tables 2 and 3**). This agrees with report on increasing S assimilation by foliar SA application (Nazar et al., 2011). However, the related mechanism and the role of the medium strength call for further investigation.

**Table 1:** The effect of combinations of salicylic acid and citric acid on mineral concentrations (fresh weight basis) in the leaf and biomass yield.

Spray treatment	Solution strength	Total dry biomass (g)	Total fresh biomass (g)	Plant height	Stem diameter (mm)	Fv/Fm	K (ppm)	Ca (ppm)	Mg (ppm)	Fe (ppm)	Mn (ppm)	Zn (ppm)	Cu (ppm)	Ni (ppm)	B (ppm)	P (ppm)	S (ppm)	Si (ppm)
CA_7_*	Full strength	1.37 abc^§^	18.81 ab	40.5 b	4 a	0.815 ab	12317 a	31975 a	8625 a	132 a	133 a	47 a	38 a	3 a	22 b	8055 a	6052 b	446 a
	Half strength	1.22 bc	16.81 bc	44.5 a	3.3 c	0.801 bc	15882 a	28107 a	6763 a	146 a	128 a	38 a	35 a	2 a	17 b	5362 a	6036 b	359 a
CA_7_SA_1_	Full strength	1.77 a	22.29 a	42.4 ab	4.2 a	0.821 a	15362 a	33482 a	8440 a	142 a	160 a	45 a	35 a	6 a	53 a	6208 a	7300 b	489 a
	Half strength	1.17 bc	15.43 bc	40 c	3.3 c	0.801 bc	16722 a	27660 a	7310 a	133 a	128 a	41 a	38 a	4 a	19 b	5008 a	6770 b	393 a
DW^†^	Full strength	1.62 ab	21.66 a	42.6 ab	3.9 ab	0.821 a	13788 a	30957 a	7550 a	138 a	139 a	46 a	38 a	9 a	21 b	6928 a	5455 b	413 a
	Half strength	1.51 abc	15.89 bc	43.2 ab	3.5 bc	0.799 bc	16372 a	31932 a	7697 a	153 a	160 a	44 a	38 a	2 a	25 ab	5243 a	8144 b	400 a
SA_1_	Full strength	1.42 abc	19.90 ab	38.2 c	4.2 a	0.817 ab	13207 a	35185 a	8220 a	154 a	137 a	51 a	41 a	6 a	12 b	8432 a	6831 b	360 a
	Half strength	1.02 c	13.52 c	38.2 c	3.6 bc	0.794 c	16872 a	30988 a	7557 a	145 a	158 a	42 a	31 a	2 a	18 b	5410 a	15087 a	374 a

^§^ Values in the same column that are followed by the same letter do not differ significantly according to Tukey’s multiple range test (*p* ≤ 0.05).

*The numbers represent concentration of foliar sprays in millimolar.

*^†^Distilled water (control).*

**Table 2:** The effect of combinations of salicylic acid and citric acid on mineral accumulation ratio in plant vs. nutrient solution (fresh weight basis).

Spray treatment	Solution strength	K	Ca	Mg	Fe	Mn	Zn	Cu	Ni	B	P	S	B
CA_7_	Full strength	264^§^ b	413 bc	325 ab	55 a	236 a	64 ab	119 a	333 a	38 ab	663 ab	48 b	90 ab
	Half strength	614 a	587 ab	413 ab	52 a	237 a	43 b	87 a	278 a	30 b	940 a	66 b	113 ab
CA_7_SA_1_	Full strength	339 b	428 bc	311 ab	60 a	290 a	61 ab	109 a	670 a	95 a	507 b	60 b	99 ab
	Half strength	646 a	579 ab	449 ab	47 a	244 a	46 ab	89 a	373 a	33 b	883 ab	75 b	124 a
DW^†^	Full strength	301 b	401 c	282 b	58 a	254 a	62 ab	113 a	963 a	37 ab	562 ab	43 b	85 ab
	Half strength	638 a	667 a	470 a	55 a	306 a	50 ab	96 a	274 a	45 ab	914 ab	90 b	125 a
SA_1_	Full strength	287 b	459 bc	308 ab	66 a	249 a	69 a	140 a	665 a	20 b	695 ab	54 b	73 b
	Half strength	655 a	648 a	464 a	52 a	295 a	47 ab	83 a	222 a	32 b	950 a	167 a	117 ab

*^§^ Values represent the accumulation ratio between concentrations of the mineral in plant tissue to the medium. Values in the same column that are followed by the same letter do not differ significantly according to Tukey’s multiple range test (*p* ≤ 0.05).*

*^†^Distilled water.*

**Table 3:** Correlation matrix between selected traits.

	Fv/Fm	Total dry biomass	Total fresh biomass	K	Ca	Mg	Fe	Mn	Zn	Cu	Ni	B	P	S
Total dry biomass	0.549**													
Total fresh biomass	0.774**	0.775**												
K	-0.423*	-0.129	-0.470*											
Ca	0.298	0.342	0.430*	-0.519**										
Mg	0.326	0.313	0.443*	-0.646**	0.733**									
Fe	0.002	0.271	0.017	0.233	0.159	-0.167								
Mn	0.090	0.071	0.014	0.073	0.463*	0.249	0.012							
Zn	0.525**	0.219	0.219	-0.335	0.255	0.162	0.255	0.226						
Cu	0.206	0.239	0.170	-0.070	0.066	0.002	0.254	-0.130	0.281					
Ni	0.496*	0.312	0.297	-0.079	-0.167	-0.087	0.292	-0.078	0.479*	0.324				
B	0.287	0.556**	0.340	0.190	0.150	0.220	-0.073	0.418*	0.035	-0.099	-0.029			
P	0.392	0.225	0.418*	-0.712**	0.637**	0.567**	-0.177	-0.114	0.375	0.206	-0.100	-0.084		
S	-0.457*	-0.246	-0.571**	0.471*	-0.156	-0.188	0.259	0.171	-0.022	-0.132	-0.077	0.084	-0.293	
Si	0.347	0.492*	0.479*	-0.047	0.028	0.264	0.048	0.190	0.031	-0.248	0.058	0.512*	-0.221	-0.064

*Correlation is significant at *p* ≤ 0.05 level.

**Correlation is significant at *p* ≤ 0.01 level.

Most of the nutrients were absorbed similarly in all treatments. However, S and B could be considered as exceptions. Boron (B) concentration and accumulation ratio was significantly higher in the CA_7_SA_1_ (7 mM CA + 1 mM SA) treatment in the full-strength medium (**Tables 1 and 2**). Bingham et al. (1970) after studies on boron absorption by excised barley root concluded that B absorption was a physical, but not a metabolic process acting in response to the B concentration gradient. On the other hand, Wildes and Neales (1971) after absorption or desorption studies reported that sliced disks of carrot and red beet absorbed B to an equilibrium internal concentration which is greater than that of the external solution, and that this accumulation of B in the tissues was inhibited by anoxia, by 2,4-dinitrophenol (DNP) and also by low temperature, suggesting active uptake. However, there are many contradictory reports in this regard as was concluded by Hu and Brown (1997). They suggested that B uptake by a particular species to be primarily determined by the B concentration in the soil solution and the rate of water uptake by the plant. However, due to dramatic differences in B concentrations among species, even when grown under similar environmental conditions and the apparent contradiction between experimental results and in field observations, they concluded that B uptake is determined by factors that are yet unknown. In our experiment that was conducted in a solution culture medium, the most important factor for B accumulation at higher concentrations could be a higher transpiration rate or presence of a physiological response pathway leading to active uptake. A significant correlation between B and silicon (Si; *p* = 0.05) which both highly depend on transpiration to accumulate is in support of the transpiration role (**Table 3**). Both of these nutrients showed a significant correlation with total dry biomass (*p* = 0.05 and *p* = 0.01 for B and Si, respectively; **Table 3**). In addition, Si content as a rough index of plant transpiration (Mayland et al., 1993) had a significant correlation with the total fresh and dry biomass (**Table 3**). However, the large increase in B content here demands for a several fold increase in transpiration to take place, which seems unreasonable.

While the difference in Si concentration was not significant among treatments (**Table 2**), its accumulation ratio was significantly higher in all treatments in the half-strength mediums. A similar trend was observed for Ca, P, K, and Mg, as well (**Table 3**). This could be due to higher transpiration in these treatments that enabled them to absorb more nutrients from a diluted medium as reported before by Cramer et al. (2008). For Si, Alternatively, the increase in Si concentration could be a result of triggering the active transport of Si in lower availability levels as suggested by Faisal et al. (2012).

Those supplied with a full-strength solution had a ticker stems and had more fresh biomass. As it could be seen in **Table 1**, change in Fv/Fm values shows that the plant had a higher photosynthetic efficiency in the full-strength solution treatment. These results indicate that this plant prefers the higher nutrient level in order to reach the maximum yield. As it is evident in **Table 3**, significant positive correlations were noticed between the Fv/Fm value and the total fresh and dry biomass (*p* = 0.01). Positive correlations were also observed between the Fv/Fm value and Zn and Ni contents of the leaf. For Zn that has a known role in the plant metabolism, this seems normal. However, for Ni, it is interesting because the known role of nickel in the plant is limited to its effect on the urease activity and thus the nitrogen metabolism (Seregin and Kozhevnikova, 2006).

Where each organic acid, when applied alone in specific concentration, proved to be not beneficial, the combination of CA and SA as foliar spray was considered more effective than applying them separately. However, in our hydroponics culture system, the increases in growth parameters were slightly better than control (distilled water, DW). This is while Mirzajani (2013) reported significant increase in growth parameters of field grown basil plants. This could be due to more complicated interactions in the soil medium that could affect on the outcome. The root exudates in soil play an important role in root function by interaction with rhizosphere microflora that has a limited role in hydroponics condition. The suggested increase in mineral absorption by elevated exudation of organic acids in response to foliar organic acids (Jafari and Hadavi, 2012) was confirmed recently by An et al. (2014), and the mediation of these exudates in mineral absorption in the soil medium is well known (Marschener, 1998; Arcand and Schneider, 2006; Bais et al., 2006).

The synergism between applied levels of CA and SA is readily notable in our results, which is consistent with the previous report. This confirms that the response to this combination takes place as an internal plant physiological response and is independent of the type of used culture medium.

We conclude that there is a pure effect by sprayed organic acids, which could be transmitted to the root and influence on the acquisition of mineral nutrients from the medium. The physiological mechanism behind this effect may not be the same for different nutrients and calls for further research. Higher B uptake in plants grown in full-strength medium and sprayed with the combination of CA_7_SA_1_ could provide a key to understand the possible mechanism of this response.

## Conflict of Interest Statement

The authors declare that the research was conducted in the absence of any commercial or financial relationships that could be construed as a potential conflict of interest.
